# Effects of HPMC on Workability and Mechanical Properties of Concrete Using Iron Tailings as Aggregates

**DOI:** 10.3390/ma14216451

**Published:** 2021-10-27

**Authors:** Xiaowei Gu, Xiaohui Li, Weifeng Zhang, Yuxin Gao, Yaning Kong, Jianping Liu, Xinlong Zhang

**Affiliations:** 1 Science and Technology Innovation Center of Smart Water and Resource Environment, Northeastern University, Shenyang 110819, China; guxiaowei@mail.neu.edu.cn (X.G.); wfzhang@stumail.neu.edu.cn (W.Z.); 2 College of Materials Science and Engineering, Chongqing University, Chongqing 400045, China; gaoyuxin@cscec.com; 3 China West Construction Academy of Building Materials, Chengdu 610015, China; kongyaning@cscec.com; 4 School of Architecture and Civil Engineering, Shenyang University of Technology, Shenyang 110870, China; pliu@sut.edu.cn; 5 Key Lab of Structures Dynamic Behaviour and Control of the Ministry of Education, Harbin Institute of Technology, Harbin 150090, China; 21b933082@stu.hit.edu.cn

**Keywords:** HPMC, iron tailings, workability, mechanical properties

## Abstract

Iron ore tailings (IOTs) are gradually used as building materials to solve the severe ecological and environmental problems caused by their massive accumulation. However, the bulk density of IOT as aggregate is too large, which seriously affects the concrete properties. Therefore, in this paper, the effect of hydroxypropyl methylcellulose (HPMC) on the workability, mechanical properties, and durability of concrete prepared from IOT recycled aggregate was studied. The action mechanism of HPMC on the workability and the mechanical properties of the IOT concrete was analyzed by mercury intrusion porosimetry (MIP) and scanning electron microscope (SEM). The results show that HPMC can effectively improve the segregation problem caused by the sinking and air entrainment of IOT aggregate and improve the crack resistance of concrete with little effect on its compressive strength and electric flux. These results are due to the air-entraining thickening effect of HPMC, which improves the slurry viscosity, hinders the sinking of aggregate, and improves the workability. At the same time, HPMC film, after concrete hardening, will bridge the slurry and aggregate through physical and chemical effects, hinder the propagation of microcracks, and improve the crack resistance.

## 1. Introduction

With the continuous development of the global social economy, the consumption of concrete as a building material is increasing rapidly at the rate of 4.4 billion tons per year [1]. More than 75% of concrete raw materials are composed of aggregates [2]. The acquisition of natural aggregates has a serious impact on the ecological environment. As a developing country, the vigorous development of infrastructure has promoted China’s rapid development. The overuse of land, minerals, water, and other non-renewable precious natural resources in the short term will damage the survival of our future generations. Currently, mineral admixtures such as fly ash (FA) [3], silica fume [4], coal bottom ash [5], and ground granulated blast furnace slag (GGBFS) [6] have been used as partial replacement of cement in concrete, which not only cuts carbon emission footprints but also reduces costs of production. Therefore, an eco-friendly production mode must be adopted to realize the sustainable development of the concrete industry.

Iron has always played an essential role in the development of modern human civilization [7]. After the iron is extracted from the iron ore by various beneficiation processes in the concentrator, a large number of iron tailings are discharged to the IOT pond for stacking [8]. China’s cumulative stockpile of tailings reached 60 billion tons, including 475 million tons of IOTs based on the 2018–2019 Chinese development report. IOTs have become one of the most critical solid wastes in the world [9]. Their massive accumulation will not only cause land pollution, poor water quality, and aggravation of dust but also threaten human health [10]. The managers of mining enterprises need to spend more resources to maintain IOT ponds every year [11]. More seriously, the existence of tailing dams is a potential safety hazard, and the human life safety events caused by the tailings dam breaks around the world are increasing every year [9]. With extensive research on the recycling of IOT, the recycled IOT is classified into four categories at home and abroad: (i) extracting valuable metals in IOT [12]; (ii) used for a backfill material [13]; (iii) turned into a building material [14,15]; (iv) reclaiming farmland on the tailings dumping ground. The large-scale application of iron tailings in concrete building materials has become the focus of all major sectors of society.

Compared with the natural aggregate, the IOT aggregate in concrete is made from the waste stone stripped from the open-pit mine through crushing and screening [16]. The IOT coarse aggregate has many edges and corners [17]. At the same time, the IOT fine aggregate has many powders during the crushing, which further increases the IOT specific surface area, increases the water demand, and leads to poor workability of IOT concrete [18].

Cellulose ethers (CEs) have been widely used in modern building products due to the function of preventing uncontrolled water loss into porous matrix [19]. HPMC, a type of CH, has been used because of its better effect on improving the workability of concrete [20]. The water retention of HPMC mainly hinders the migration of water molecules between pores through the action of internal groups. Pourchez et al. reported that CE slows down the hydration process of cement, which helps to reduce the loss of water [21]. Brumaud et al. revealed how CE slows down the cement hydration process [22]. Weyer et al. showed that it is necessary to control the CE content [23]. Only adding an appropriate amount of CE can slow down the hydration of cement.

Until now, researchers have studied the mechanism of different kinds of CEs in paste and mortar, but there are few reports of HPMC on concrete, especially the concrete used by IOTs as an aggregate. This paper focuses on the effect of HPMC on the workability of full IOT aggregate concrete. Moreover, the influence mechanism of HPMC on the mechanical properties of IOT concrete was also clarified.

## 2. Materials and Methods

### 2.1. Raw Materials

Cement (P.O. 42.5) is the main binder produced by Benxi Yuntian Cement Co., Ltd. (Benxi, China) according to GB/T 175-2007 [24]. The physical and chemical properties of cement are presented in Table 1.

**Figure 1:**
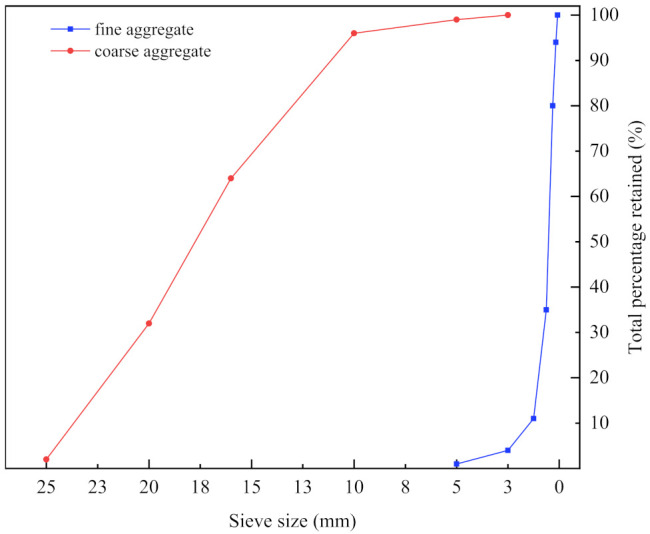
The gradation compositions of coarse and fine aggregates.

The coarse and fine aggregates are formed by the IOT waste rock mechanism. The particle size of the coarse aggregate is 5–20 mm, while the fineness modulus of the fine aggregate is 2.2. The gradation compositions of coarse and fine aggregates are presented in Figure 1, respectively. Table 2 displays the aggregates’ basic properties. The coarse and fine aggregates were evaluated according to GB/T 14685-2011 and GB/T 14684-2011, respectively. Polycarboxylic acid high-performance water reducer (water reduction rate: 34%) was used to ameliorate the workability of concrete supplied by the Changyuan admixture technology Co., Ltd. (Jilin, China). The NDJ viscosity of HPMC was 196,000 mPa·s. and was purchased from Huzhou, Zhejiang.

### 2.2. Mixture Proportions

The mixture design of IOT concrete in this paper is shown in Table 3. In HPMC0, the HPMC is not added in concrete. In HPMC1, the HPMC is added into concrete. HPMC and an appropriate amount of water were added to the agitator cup and stirred at the speed of 3000 rpm for 180 s. HPMC is 0.1% by mass of cement [26]. The characteristic of fly ash admixture is shown in Table 4.

### 2.3. Specimen Preparation

The SJD60 concrete horizontal-axle mixer was selected for concrete preparation. The cement and fly ash were dry mixed for one minute, the aggregate was added for another minute, and water and SPs were added for another two minutes. Specimens of 100 mm cubes, 150 mm cubes, and 100 × 400 mm prisms were prepared for each concrete mixture. The 100 mm and 150 mm cubes were used to determine the compressive strength and splitting tensile strength tests. The prisms were used to evaluate the flexural strength test. The permeability test took a 50 mm thick round pie sample from a 100 × 200 mm cylinder. The samples were cured under ambient conditions for 1 d, demolded, and further cured in a standard curing tank at a temperature of 20 ± 2 °C and humidity of more than 95% until the aging.

### 2.4. Test Methods

#### 2.4.1. Fresh Properties

The slump, expansion, and entrained air content of the IOT concrete were measured. The slump and expansion tests were conducted by ASTM C143-15a [27]. The entrained air content of concrete mixtures was measured using the water column method according to the British Standard EN 12350-7 [28]. For the slump and entrained air content experiments, each group of experiments obtained the average value and the result by measuring three values. The average of the two outermost expansion diameters was taken as the final result for the expansion experiment.

#### 2.4.2. Mechanical Properties

The mechanical properties of IOT concrete were determined using a universal testing machine with a loading capacity of 2000 kN following the Chinese Standard GB/T50081-2002 [29]. The loading rates applied in the compressive, splitting tensile, and flexural strengths were 0.6 MPa/s, 0.06 MPa/s, and 0.06 MPa/s, respectively. The flexural strength was measured with a four-point bending test. The results of the above three strength tests were determined by the average value of three measured results.

#### 2.4.3. Permeability Test

To determine the chloride ions charge in the concrete specimen, the Chloride penetration tests were conducted according to ASTM C1202 [30]. The cylindrical sample, 100 × 200 mm, was cut into 100 two-part pieces of 100 × 100 mm. We then took 50 mm thick samples from the two parts respectively and used the surface of the first cut as the test surface. After cutting, the sample shall be vacuum water retained. The cathode solution is 3% NaCl solution, and the anode solution is 0.3 mol/l NaOH solution. The DC power supply for six hours can stably output 60 V voltage, the accuracy can meet the requirements of 0.1 V, and the current is in the range of 0–10 A. Use a data logger to record the total charge passed in 6 h every 30 min. Table 5 shows the evaluation of chloride ion permeability grade.

#### 2.4.4. Mercury Intrusion Porosimetry

The mercury intrusion porosimetry (MIP) testing instrument is AutoPore IV 9500, produced by the Micrometrics Instrument Corporation. The relationship between the applied pressure and the cylindrical pore diameter is described by the Washburn equation [31]. The total porosity and the pore size distribution could be obtained from MIP. The threshold pore diameter above which minimal intrusion occurs is still appropriate as a comparative index for the connectivity of different samples [32]. The threshold pore diameter is closely related to the permeability and diffusivity of cementitious materials and could be considered an indicator of material durability [33]. After curing to the corresponding age, the cube was sliced and soaked in absolute ethanol for seven days to terminate hydration. The samples were then dried in a 50 °C oven for two days.

#### 2.4.5. Scanning Electron Microscopy-Backscattered Electron-Image Analysis (SEM-BSE-IA)

The slices were cut, immersed in isopropanol, and dried in an oven. Then, the slices were immersed in epoxy resin under vacuum for about 30 min and placed for hardening for one day. The hardened sample was first polished on sandpaper (320#) so that the sample surface was completely exposed, and the sample edge could be seen under the irradiation of a fluorescent lamp. Then, the samples were polished to 1 μm until the sample surface was smooth and flat on the polishing machine, using diamond suspension as the polishing solution and gasoline as a lubricant. The sandpaper grades used ranged from 800#, 1000#, 1200#, 1500#, 2000#, 2500#, to 4000#. According to the sample surface conditions and each grinding condition, the grinding time ranged from 20 to 60 s. In order to avoid the influence of residual diamond impurities and improve the efficiency of sample preparation, the samples need to be cleaned with ultrasonic in isopropyl alcohol solution during polishing. The polishing disc needed to be cleaned with a special detergent and brush. After polishing, the samples were stored in a vacuum dryer for 2 days to remove the residual organic solvent and avoid polluting the probe inside the scanning electron microscope (SEM). The samples were sprayed with carbon to form a carbon layer with a thickness of about 10 nm on the surface before the SEM test. The backscattered electron (BSE) images were obtained by FEI FEG quanta 650 field emission environmental scanning electron microscope with an accelerating voltage of 20 kV and a beam spot diameter of 3.0 nm.

BSE was used to evaluate the properties of ITZ, and the image analysis was carried out to quantify the microstructure. The ImageJ software was used to remove the aggregate. For image binarization, a 5 μm strip was used. The proportion of the object image was obtained by dividing the number of pixels in the gray range of the object image by the total number of pixels in the strip. The multiples and resolutions of the images used were consistent.

## 3. Results and Discussion

### 3.1. Workability

The iron ore tailings are generated by the waste rock mechanism stripped from the open-pit mine. Compared with ordinary natural aggregate, the IOT has a larger bulk density due to the iron content inside, leading to the natural sinking and difficulty of mortar binding with the aggregate [16]. When IOT is used as a fine aggregate, the specific surface area is large, and the water absorption is high due to the internal stone powder, which worsens the workability of the IOT concrete. The fineness modulus of raw materials used to prepare concrete is positively correlated with the workability of concrete [34].

**Figure 2:**
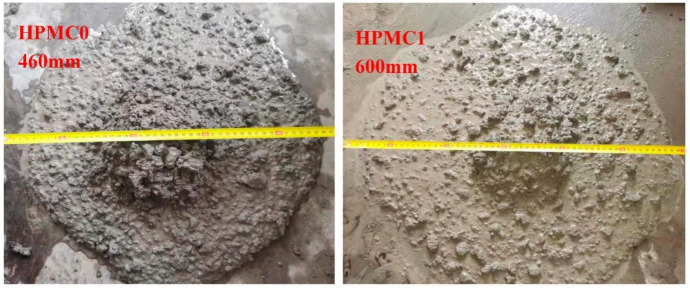
Changes of concrete workability before and after adding HPMC.

The addition of HPMC improves the fluidity of concrete, which is mainly due to the action of HPMC’s chemical groups to improve the water retention effect of concrete so that more mortar can well wrap the aggregate (Figure 2, Table 6). As the entrained air content experiment results show, in addition to ameliorating the water retention of concrete, the HPMC also introduces a large number of bubbles, resulting in a significant increase in the entrained air content of concrete from 1.6% to 4.5%. Chen [26] also found that the addition of HPMC can effectively improve the water retention performance of concrete, resulting in significant improvement in workability.

### 3.2. Mechanical Properties

#### 3.2.1. Compressive Strength

**Figure 3:**
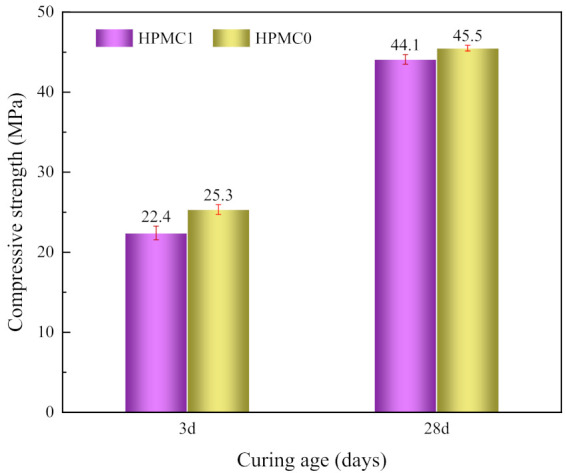
Development of compressive strength at different curing ages.

The results of compressive strength tests of IOT concrete with and without HPMC are presented in Figure 3. The compressive strength of HPMC1 was lower than that of HPMC0, regardless of curing ages. The reduction rate at 3 and 28 days was 11.5% and 3.1%, respectively. The macro strength of the concrete mainly depends on the micropore structure distribution and porosity [35]. Although the addition of HPMC makes the water retention effect of concrete better, it increases the internal porosity while the compressive strength is decreased. Furthermore, when the concrete is mixed with water, the HPMC latex particles are adsorbed on the cement surface to generate a latex film, reducing the cement hydration and compressive strength [20]. The particles’ pore plugging induces a decrease in the water transport properties of the cement matrix [36]. With the continuous hydration, the decreased range of compressive strength is decreasing, indicating that the increasing hydration products are making up for the loss of compressive strength caused by HPMC. This corresponds to the above experimental results of entrained air content, which proves that introducing many bubbles weakens the compressive strength of the IOT.

#### 3.2.2. Splitting Tensile Strength

**Figure 4:**
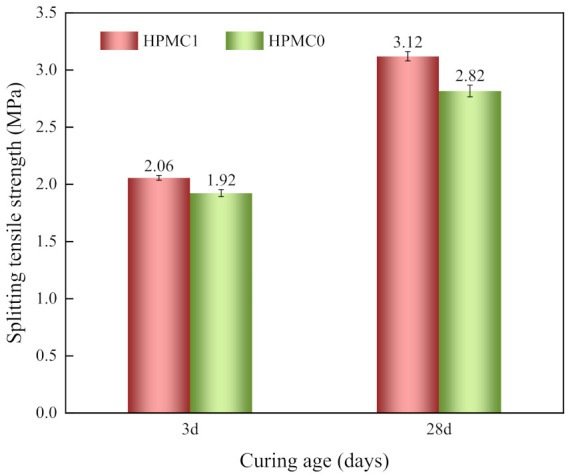
Splitting tensile strength of concrete with increasing age.

The splitting tensile strength test was presented in Figure 4. The results indicate that the splitting tensile strength increased when adding HPMC at all curing ages. The growth rate of splitting tensile strength at 3 and 28 days was 7.3% and 10.6%, respectively. When HPMC is added, the methoxy and hydroxypropyl groups on the molecular chains react with Ca^2+^ and Al^3+^ to form a viscous gel and fill in the concrete internal void, playing a role of flexible reinforcement, thereby improving the compactness of concrete and increasing the splitting tensile strength. Moreover, smaller IOT powder enhances the bond between the aggregate and the matrix, improving the splitting tensile strength [37].

#### 3.2.3. Flexural Strength

**Figure 5:**
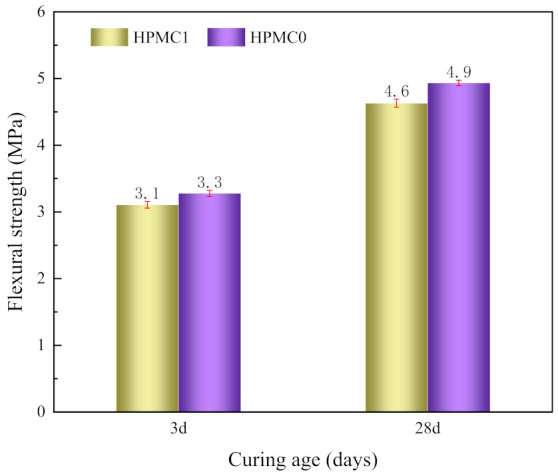
Development of flexural strength of IOT concrete at different curing ages.

The flexural strength test results of IOT concrete with and without HPMC are displayed in Figure 5. The flexural strength of HPMC1 was lower than that of HPMC0 at 3 and 28 days curing age. The reduction range of flexural strength was 6.06% and 6.12%, respectively. It can be observed that the flexural strength is not sensitive to the addition of HPMC. The addition of HPMC improves the water retention effect of IOT concrete, but the internal porosity also increases, resulting in reduced flexural strength. HPMC latex particles form latex film by adsorbing on the cement surface, reducing the cement hydration and flexural strength.

### 3.3. Chloride Penetration Test

**Figure 6:**
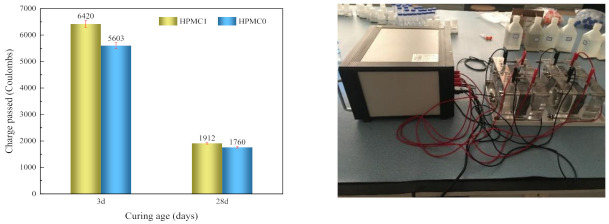
The charge passed of concrete with and without HPMC at different curing ages.

The chloride ion flux results for HPMC0 and HPMC1 are displayed in Figure 6. The HPMC1 shows higher charges passed compared to HPMC0 at 3 and 28 days. The charges passed were greater than 4000 C at three days, which was classified as high (seen in Table 5) according to ASTM C1202 standard. At the initial stage of hydration, a large number of bubbles were introduced by HPMC, increasing the internal porosity and resulting in poor resistance to chloride ion permeability. The coulomb charge in HPMC0 and HPMC1 specimens reduced significantly after 28 days. The charges passed were within the 1000–2000 C range, which was classified as low. With the continuous hydration reaction, more hydration products fill the internal pores, which reduces the porosity, optimizes the pore size distribution, and reduces the chloride ion electric flux significantly. Pierre [38] reported that HPMC would plug part of the cement paste porosity, which increases the apparent viscosity and leads to a substantial decrease in the material’s apparent permeability. It has also been shown that the retardation mechanism results mainly from the adsorption of HPMC onto the surface of cement grains [39]. The addition of HPMC increases the entrained air content of concrete, and the introduction of many bubbles increases the internal pores, which do not block the passage of chloride ions.

### 3.4. MIP

**Figure 7:**
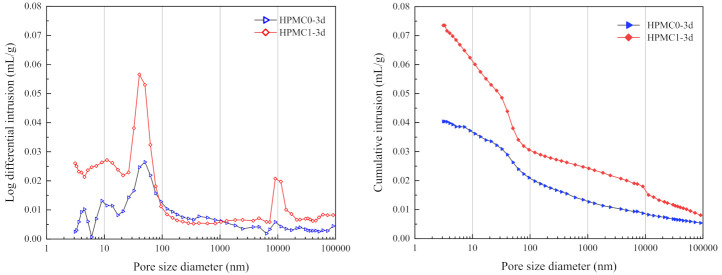
The pore structure of HPMC0 and HPMC1 at 3 days.

**Figure 8:**
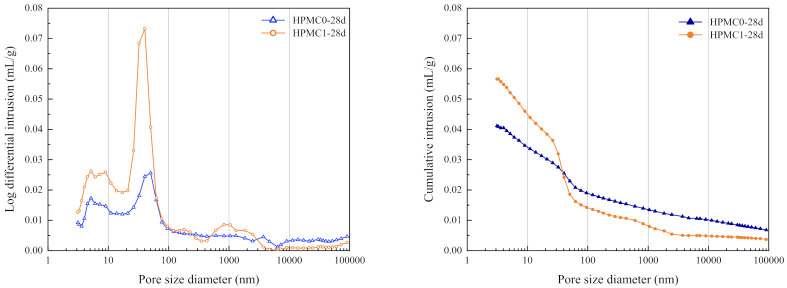
The pore structure of HPMC0 and HPMC1 at 28 days.

The differential pore size distribution and cumulative porosity of HPMC0 and HPMC1 are shown in Figure 7 and Figure 8. The harmful pores and total porosity of HPMC0 and HPMC1 at 3 and 28 days are as shown in Table 7. The addition of HPMC can significantly increase the pore volume and the total porosity after curing for three days. The addition of HPMC introduces many bubbles, which increases the entrained air content and the porosity of IOT concrete. Figure 7 demonstrated that the cumulative pore volume increased from 0.025 mL/g to 0.06 mL/g. As generally accepted, when the pores inside the concrete increase more than 20 nm or even close to 100 nm, they can reduce the properties of concrete, e.g., mechanical properties, permeability, and durability. Hence, when adding HPMC, the number of harmful pores (pore size in the range of 50 nm to 200 nm) showed a slight increase, which means that its compressive strength, flexural strength, and chloride resistance were reduced [40].

After curing for 28 days, the harmful pores and the total porosity for HPMC0 and HPMC1 have decreased compared to those cured for only three days (as shown in Table 7). The total porosity from 3 days to 28 days was reduced by 0.18% and 2.81%, respectively, indicating that the hydration products filled the voids and refined the pore structure with the increase of curing age.

The MIP test results confirm the overall performance; as the entrained air content of concrete increases, the compressive and flexural strength decreases, and the chloride ion electric flux increases after adding HPMC.

### 3.5. SEM-BSE-IA

Under the condition of constant stress, the different elastic and shear moduli of aggregate and matrix lead to the different strains. Currently, ITZ plays a bridging role between the aggregate and the matrix. Even if each phase in concrete has a high hardness, if ITZ can not effectively transfer stress, the concrete strength will be reduced [41]. Therefore, ITZ is considered the weakest link of concrete strength as it leads to the strength of concrete being lower than that of aggregate and cement [42].

**Figure 9:**
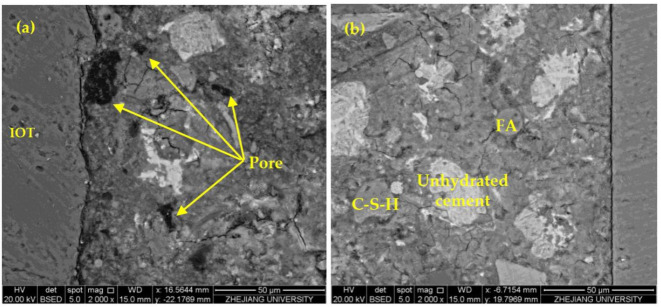
The interface transition zone (ITZ) of HPMC1 (**a**) and HPMC0 (**b**) at 28 days.

Microcracks and high porosity in ITZ are the key factors affecting the mechanical properties of concrete. Large size CH will weaken the van der Waals force, decreasing the CH interlayer bonding ability, which is not conducive to the mechanical properties of concrete [43]. After adding HPMC, as Figure 9 shows, the ITZ contained many pores, the increase of porosity made the combination of aggregate and matrix relatively loose, slender ettringite was generated, and large CH crystals were enriched to form the preferred orientation. The ITZ without HPMC was fairly dense, and the enrichment volume of CH crystals formed near the aggregate was small. The SEM-BSE-IA test results confirmed that the addition of HPMC increased the pores in ITZ, loosened the structure, and worsened the mechanical properties.

**Figure 10:**
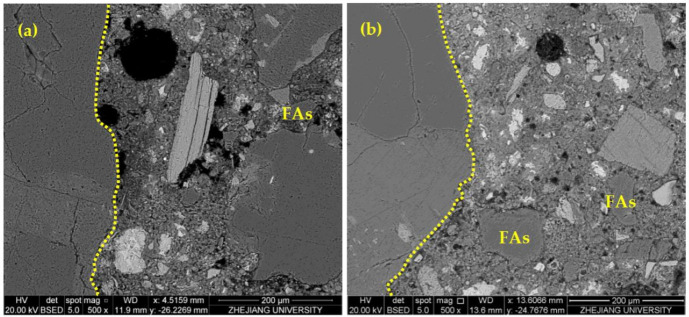
Quantitative calculation of the interfacial transition zone: HPMC1 (**a**) and HPMC0 (**b**) at 28 days.

Figure 10 analyzed the porosity and unhydrated rate in the ITZ of HPMC0 and HPMC1, and the results are shown in Table 8 and Table 9. In freshly mixed concrete, the water film is formed on the surface of the large aggregate particles so that the water–cement ratio in the ITZ is much larger than that of the cement–mortar matrix. The water–cement ratio is significant, and there are plate-like calcium hydroxide crystals and the acicular column ettringite enrichment near the aggregate surface, forming a framework with more pores than the cement–mortar matrix. Therefore, the closer to the aggregate, the higher the porosity of the ITZ, the higher the degree of hydration, and the lower the non-hydration rate.

## 4. Conclusions

In this study, the effect of HPMC on the workability of IOT concrete was studied. Moreover, the influence mechanism of HPMC on the mechanical properties of IOT concrete was further clarified. Based on the experimental results, several conclusions can be drawn:

(1) Due to the iron content in tailings aggregate, the density is relatively large, which leads to the aggregate sink and separation. The surface activation of HPMC can induce air, retain water and thicken to improve the viscosity of mortar and aggregate, prevent tailings aggregate from sinking, and improve the workability of concrete.
(2) Although HPMC can improve workability, it is not conducive to the development of compressive and flexural strength. However, it can improve the splitting tensile strength, which shows that the addition of HPMC can improve crack resistance.
(3) When the concrete is hardened, the air entrainment of HPMC will form pores and reduce the compressive strength and flexural strength. At the same time, HPMC reacts with Ca^2+^ and Al^3+^ to form membrane structure, which plays a bridging role, hinders the propagation of microcracks, improves the splitting tensile strength of concrete, and then ameliorates the crack resistance of concrete.
(4) The ITZ of ordinary C30 concrete is the key to affecting the concrete’s strength and its weakest link. The addition of HPMC increases more bubbles in the ITZ and reduces the compactness, which is not conducive to the development of strength. However, the final strength grade is consistent with that without HPMC. The chloride ion flux is about 1800, which does not affect the durability. Therefore, it is an effective measure to improve the workability of IOT concrete by adding HPMC.

## Figures and Tables

**Table 1:** Physical and chemical properties of cement.

Chemical Composition	(wt.%)	Physical Properties	Value
SiO_2_	28.16	Specific surface area (m^2^/kg)	400
Al_2_O_3_	7.44	Water requirement of consistency/%	26.0
Fe_x_O_y_ [25]	2.76	Initial setting/min	185
CaO	54.86	Final setting/min	260
Na_2_O	0.03	3 d flexural strength/MPa	4.1
MgO	4.36	28 d flexural strength/MPa	8.2
SO_3_	2.24	3 d compressive strength/MPa	21.0
Loss	2.67	28 d compressive strength/MPa	49.4

**Table 2:** The basic properties of aggregates.

	Coarse Aggregate	Fine Aggregate
**Apparent density (kg/m^3^)**	2630	2590
**Bulk density (kg/m^3^)**	1490	1540
**Sediment percentage/%**	0.3	2.4
**Clay lump/%**	0	0.8
**Void ratio/%**	43	41
**Strength crushing index/%**	7.4	/
**Needle flake particle content/%**	3	/

**Table 3:** Mixture proportions.

Symbol	C (kg/m^3^)	FA (kg/m^3^)	FAs (kg/m^3^)	CAs (kg/m^3^)	Water (kg/m^3^)	SPs (kg/m^3^)	HPMC (kg/m^3^)	w/b
HPMC0	380	40	875	945	165	4	/	0.4
HPMC1	380	40	875	945	165	4	0.38	0.4

Note: Cement (C); Fly ash (FA); Fine Aggregates (FAs); Coarse Aggregates (CSa); Superplasticisers (SPs); Hydroxypropyl methyl cellulose (HPMC); water to binder (w/b).

**Table 4:** The characteristic of fly ash admixture.

Test Items	Detection Result
Water demand ratio/%	87
Loss on ignition/%	1.21
Water content/%	0.5
Sulfur trioxide/%	0.23
Free calcium oxide content/%	0.52
SiO_2_/wt%	60.1
Al_2_O_3_/wt%	25.1
Fe_2_O_3_/wt%	6.7

**Table 5:** Evaluation of chloride ion permeability grade.

Chloride Permeability	Charge (Coulomb)	Typical Concrete
High	>4000	High w/c ratio (>0.6)
Moderate	2000–4000	Moderate w/c ratio (0.4–0.5)
Low	1000–2000	Low w/c ratio (<0.4)
Very low	100–1000	Latex-modified concrete, internally sealed concrete
Negligible	<100	Polymer impregnated concrete, polymer concrete

**Table 6:** Multiple indexes of concrete workability.

Symbol	Slump (mm)	Expansion (mm)	Entrained Air Content (%)
HPMC0	220	460	1.6
HPMC1	240	600	4.5

**Table 7:** Harmful pores and total porosity of HPMC0 and HPMC1.

Symbol	Harmful Pores (50–200 nm) (mL/g)	Total Porosity (%)
HPMC0-3d	0.105	9.51
HPMC1-3d	0.137	15.25
HPMC0-28d	0.077	9.33
HPMC1-28d	0.097	12.44

**Table 8:** BSE quantitative calculation of HPMC0.

Distance from Aggregate/μm	Porosity/%	Unhydrated Rate/%
5	43.27	15.96
10	30.24	34.95
15	20.33	44.17
20	13.95	52.68

**Table 9:** BSE quantitative calculation of HPMC1.

Distance from Aggregate/μm	Porosity/%	Unhydrated Rate/%
5	59.44	15.50
10	53.18	19.55
15	52.85	20.07
20	47.85	24.06

## Data Availability

Not applicable.
